# Big Data Sensors of Organic Advocacy: The Case of Leonardo DiCaprio and Climate Change

**DOI:** 10.1371/journal.pone.0159885

**Published:** 2016-08-02

**Authors:** Eric C. Leas, Benjamin M. Althouse, Mark Dredze, Nick Obradovich, James H. Fowler, Seth M. Noar, Jon-Patrick Allem, John W. Ayers

**Affiliations:** 1 University of California San Diego School of Medicine, San Diego, CA, United States of America; 2 Institute for Disease Modeling, Bellevue, WA, United States of America; 3 University of Washington, Seattle, WA, United States of America; 4 Santa Fe Institute, Santa Fe, NM, United States of America; 5 New Mexico State University, Las Cruces, NM, United States of America; 6 Human Language Technology Center of Excellence, Johns Hopkins University, Baltimore, MD, United States of America; 7 Bloomberg LP, New York, New York, United States of America; 8 Scripps Institution of Oceanography, University of California San Diego, San Diego, CA, United States of America; 9 Department of Political Science, University of California San Diego, San Diego, CA, United States of America; 10 School of Media and Journalism, University of North Carolina, Chapel Hill, NC, United States of America; 11 Lineberger Comprehensive Cancer Center, University of North Carolina, Chapel Hill, NC, United States of America; 12 Keck School of Medicine, University of Southern California, Los Angeles, CA, United States of America; 13 Graduate School of Public Health, San Diego State University, San Diego, CA, United States of America; New York City Department of Health and Mental Hygiene, UNITED STATES

## Abstract

The strategies that experts have used to share information about social causes have historically been top-down, meaning the most influential messages are believed to come from planned events and campaigns. However, more people are independently engaging with social causes today than ever before, in part because online platforms allow them to instantaneously seek, create, and share information. In some cases this “organic advocacy” may rival or even eclipse top-down strategies. Big data analytics make it possible to rapidly detect public engagement with social causes by analyzing the same platforms from which organic advocacy spreads. To demonstrate this claim we evaluated how Leonardo DiCaprio’s 2016 Oscar acceptance speech citing climate change motivated global English language news (Bloomberg Terminal news archives), social media (Twitter postings) and information seeking (Google searches) about climate change. Despite an insignificant increase in traditional news coverage (54%; 95%CI: -144 to 247), tweets including the terms “climate change” or “global warming” reached record highs, increasing 636% (95%CI: 573–699) with more than 250,000 tweets the day DiCaprio spoke. In practical terms the “DiCaprio effect” surpassed the daily average effect of the 2015 Conference of the Parties (COP) and the Earth Day effect by a factor of 3.2 and 5.3, respectively. At the same time, Google searches for “climate change” or “global warming” increased 261% (95%CI, 186–335) and 210% (95%CI 149–272) the day DiCaprio spoke and remained higher for 4 more days, representing 104,190 and 216,490 searches. This increase was 3.8 and 4.3 times larger than the increases observed during COP’s daily average or on Earth Day. Searches were closely linked to content from Dicaprio’s speech (e.g., “hottest year”), as unmentioned content did not have search increases (e.g., “electric car”). Because these data are freely available in real time our analytical strategy provides substantial lead time for experts to detect and participate in organic advocacy while an issue is salient. Our study demonstrates new opportunities to detect and aid agents of change and advances our understanding of communication in the 21st century media landscape.

## Introduction

On February 28^th^, 2016 Leonardo DiCaprio used his Oscar acceptance speech to advocate for action on climate change to 34.5 million live viewers **(Box 1)** [1]. DiCaprio is not alone in championing social causes, especially climate change advocacy [2,3]. Recent trends suggest that a large number of non-celebrities are also conscious of social and environmental causes, with 81% of global consumers claiming they would be willing to purchase/consume fewer products to preserve natural resources [4]. Moreover, the Web 2.0 era has democratized advocacy. Unlike ever before, the public can seek, create, and share information instantaneously online [5]. We call this phenomenon “organic advocacy” since it is often led by the public and occurs outside the context of expert-led events or campaigns.

**Box 1**

“And lastly, I just want to say this, making The Revenant was about man's relationship to the natural world—the world that we collectively felt in 2015 [w]as the hottest year in recorded history. Our production had to move to the southernmost tip of this planet just to be able to find snow. Climate change is real, it is happening right now, it is the most urgent threat facing our entire species, and we need to work collectively together and stop procrastinating. We need to support leaders around the world who do not speak for the big polluters or the big corporations, but who speak for all of humanity, for the indigenous peoples of the world, for the billions and billions of underprivileged people who will be most affected by this, for our children's children, and for those people out there whose voices have been drowned out by the politics of greed.” ~*Transcript from Leonardo Dicaprio’s Oscar Acceptance Speech*

Population-level engagement with a campaign or organized event is typically assessed using surveys, but surveys are ill equipped to study discrete occurrences of organic advocacy, such as DiCaprio’s speech. Surveys are costly and typically do not have sampling frames that allow for the necessary baseline measures to demonstrate deviations from historical trends. These barriers alone may explain why organic advocacy has not been well-studied or integrated into expert communication strategies. Significant breakthroughs in big data are eliminating some of these barriers. Online footprints continuously shadow how the public engages with and seeks information [6,7], including with environmental issues and climate change [8,9]. By harnessing these novel data streams, we can directly observe how the public engaged with DiCaprio’s call-to-action. And because these data are available in real time, our strategy can be taken to the field to inform how experts respond to advocacy as it is happening. Specifically, we explored how DiCaprio’s message resonated on (a) traditional news and social media, and (b) how the public concurrently sought information on how to respond online. We also compared DiCaprio’s speech to other organized climate change events, such as Earth Day, to contrast the potential impacts of organic advocacy to planned advocacy.

## Materials and Methods

Evaluating changes in traditional news and social media relied on daily trends for global news articles from the Bloomberg Terminal using the {NT GO} function (bloomberg.com/professional) and postings on Twitter (dev.twitter.com/streaming/overview) that included the phrases “climate change” or “global warming” from January 1, 2011 through March 9, 2016. News was plotted on a relative scale (the proportion of all news including the key terms) and Twitter on an absolute scale (all English language tweets including the key terms) since it was only available in this format.

To evaluate how the public sought information we monitored all English language Google searches (google.com/trends), taking into account the specific topics the public sought information on, by hour for February 25, 2016 through March 9, 2016. Google searches were divided between those primarily investigating the claims made by DiCaprio, including the exact terms “climate change”, “global warming,” “hottest year,” and “indigenous,” versus those investigating topics that are related to climate change, but were not mentioned by DiCaprio, including the exact terms “solar power,” “electric car,” “sea level rise,” and “carbon tax.” Searches were plotted on a relative search volume (the proportion of searches including the key terms relative to all searches scaled to a 0 to 100 scale where 100 is the highest search proportion) and absolute volumes were estimated using Google Adwords (google.com/adwords).

Our study design was quasi-experimental, deriving a single estimate of the “DiCaprio effect” by comparing observed news, Twitter, and search volumes around DiCaprio’s speech to a counterfactual estimate representing expected news, Twitter, and search volumes had DiCaprio not advocated for climate change during the Oscars [10]. The observed volumes were derived from the time series. The counterfactual volumes were derived from an autoregressive integrated moving average (ARIMA) model, with components estimated using the algorithm developed by Hyndman and Khandakar [11]. This model used historical data to estimate expected volumes for the time period after DiCaprio’s speech. These models are robust to the most well-known biases, including recurring periodicities and trending in the data, such as how the daily volume of tweets may be growing as Twitter’s user base grows. The ratio of observed and counterfactual volumes were calculated so as to represent the “effect” as a percent increase with confidence bounds (alpha = 0.05) estimated by using 10,000 random draws from the multivariate normal sampling distribution with mean equal to the maximum-likelihood point estimates, and variance equal to the variance-covariance matrix [12]. We fitted additional models to describe the durability of the “DiCaprio effect” and describe variations by hour or day.

To provide a practical comparison, we also studied the most recent United Nations Conference of the Parties (COP), an international brokerage of a global climate change agreement, and the 45^th^ Earth Day celebration. This analysis relied on the same data trends and analytic approaches used to study the DiCaprio effect. In summary, each time trend was analyzed and the observed volumes were derived from each respective time series, including November 30, 2015 though December 12, 2015 for COP and April 22, 2015 for Earth Day, and the counterfactual volumes were derived from forecasts using the Hyndman and Khandakar method [11]. The ratios of observed and counterfactual volumes were used to compute the COP effect and Earth Day effect for each data type. Because the COP occurs over several days we computed the summary COP effect using the daily average for all days during COP. The resulting COP effect and Earth Day effect was then compared to the DiCaprio effect (e.g., Dicaprio effect divided by the summary COP effect) with statistical tests estimated by using 10,000 random draws from the multivariate normal sampling distribution with mean equal to the maximum-likelihood point estimates, and variance equal to the variance-covariance matrix [12].

All analyses relied on anonymized data and adhere to the terms and conditions, terms of use, and privacy policies of Bloomberg LP, Google, and Twitter. All analyses were computed using R Ver. 3.2.1

## Results

**Fig 1:**
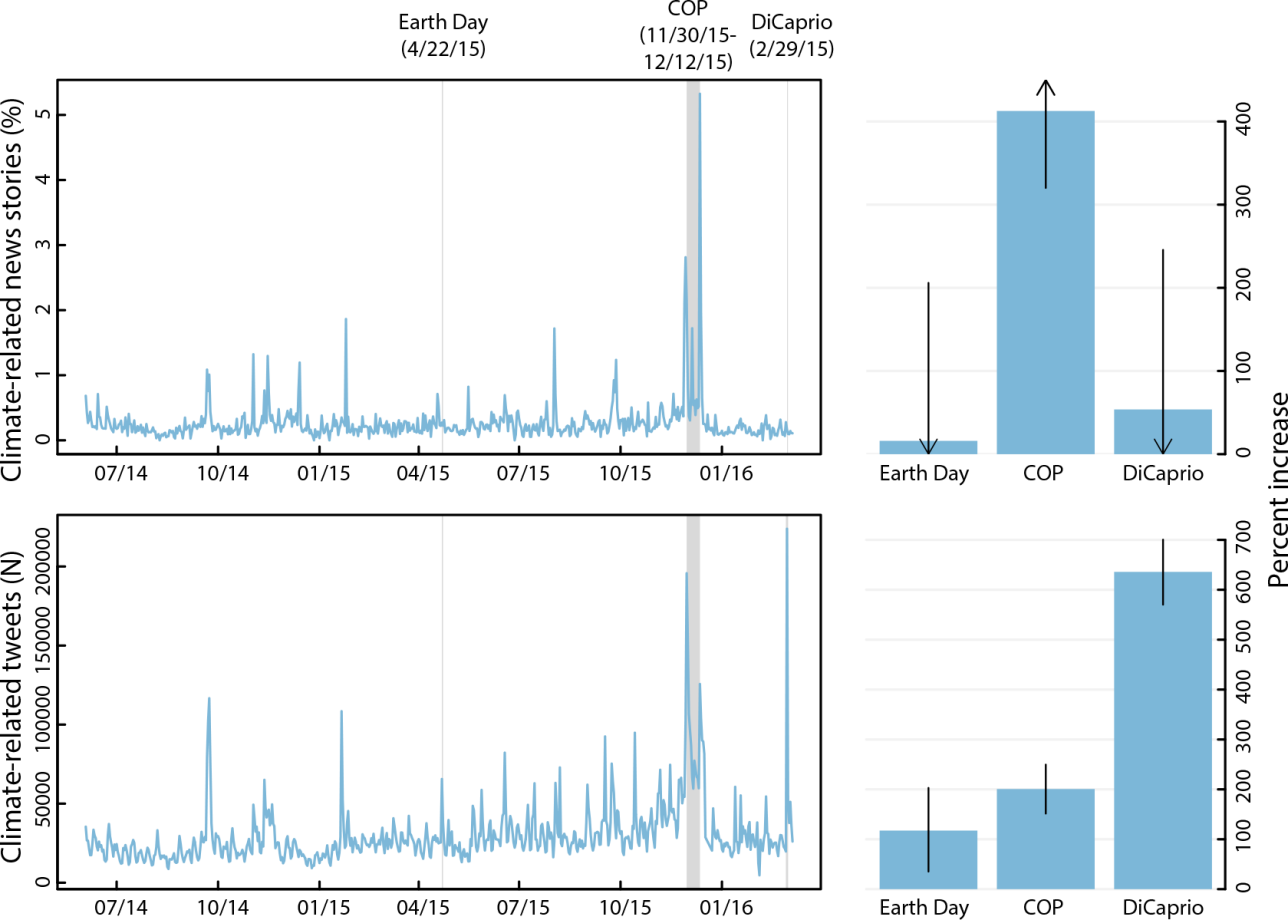
News articles and Twitter postings for “climate change” or “global warming” Time trends show daily trends for news articles and Twitter postings that included the terms “climate change” or “global warming.” Bar plots show estimated increases in daily volumes for news articles and Twitter postings the day of DiCaprio’s Oscar speech, the daily average during the 2015 Conference of the Parties meeting, and 2015 Earth Day, comparing the observed volume to an empirical expectation of the counterfactual volume derived from forecasts using historical trends. Arrows indicate that the confidence interval bound continues off the plotted image.

Social media engagement on climate change spiked after DiCaprio’s speech even though news coverage of climate change did not statistically significantly increase (54%; 95%CI: -144 to 247). Tweets mentioning climate change or global warming were 636% (95%CI: 573–699) higher than expected the day DiCaprio spoke **(Fig 1)**.

To put these trends in perspective, the response to DiCaprio’s speech on Twitter was 5.3 times larger (p = 0.003) than Earth Day (Earth Day = 117%; 95%CI: 35–202) and 3.2 times larger (p < 0.0001) than the daily average during the most recent COP (COP = 200%; 95%CI: 149–251). Moreover, the number of tweets including the phrases “climate change” or “global warming” on the day of DiCaprio’s speech were at the highest recorded value in our database with more than 250,000 tweets on that day.

**Fig 2:**
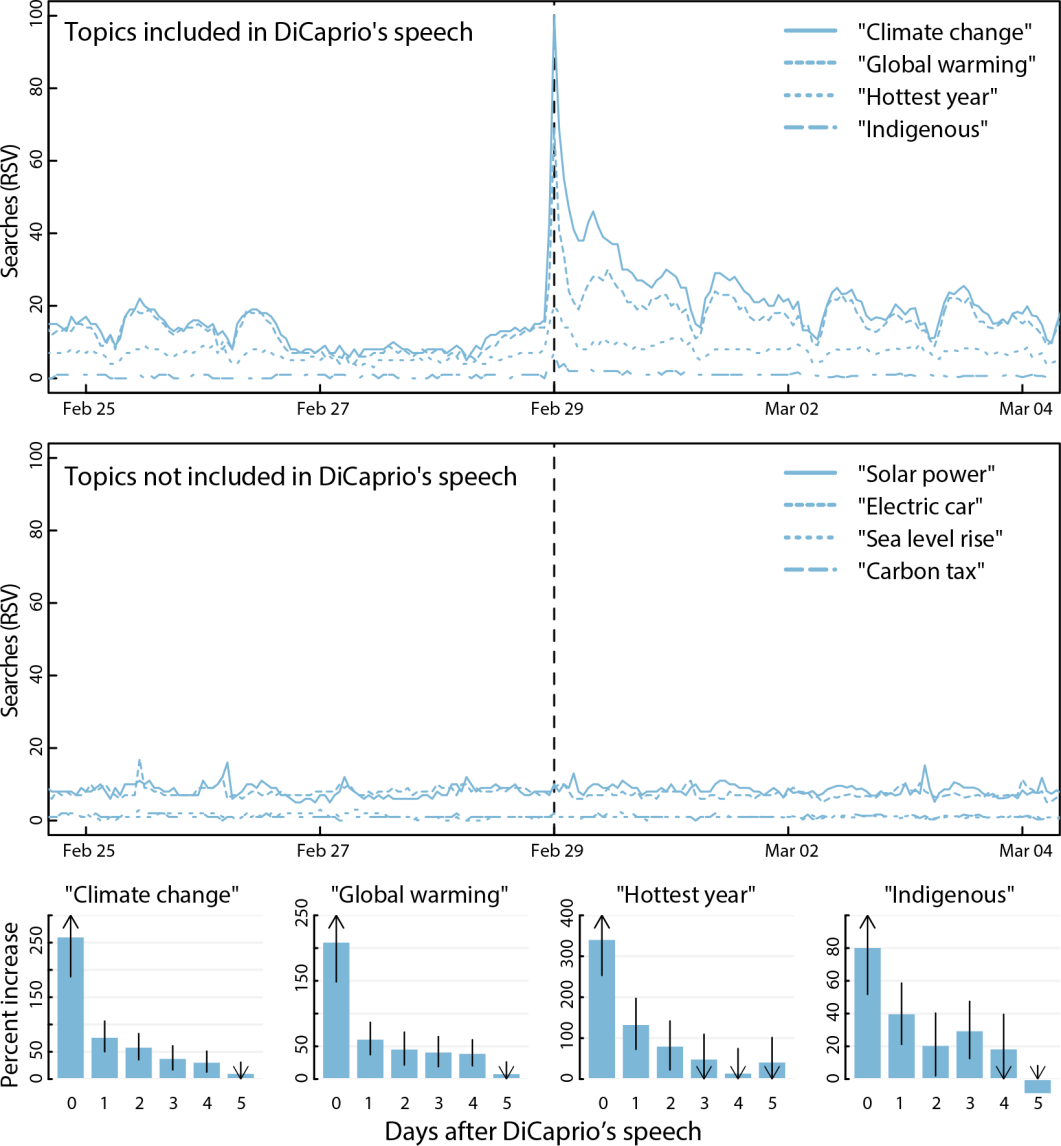
Hourly Google searches around DiCaprio’s Oscar speech. Time trends show Google search volumes for the referenced focal terms, clustered according to those included (versus not included) in DiCaprio’s speech. Bar plots show estimated increases in each focal term, with estimates showing the day of DiCaprio’s speech (0) and subsequent days labeled as 1 through 5. Estimates were derived by comparing the observed volume to an empirical expectation of the counterfactual volume derived from forecasts using historical trends.

Increased engagement on social media co-occurred with increased information seeking for content from DiCaprio’s speech (**Fig 2**). Google searches for “climate change” increased immediately the hour DiCaprio spoke, rising 261% (95%CI: 186–335) the day of, 78% (95%CI: 51–105) the day after, and remained significantly higher 4 days later (39%; 95%CI: 17–61); accounting for 104,190 total searches. “Global warming” searches were 210% (95%CI: 149–272) higher the day of and still 42% (95%CI: 19–65) higher 4 days later, representing 216,490 total searches. In relative terms, the day DiCaprio spoke these searches were 3.8 (p = 0.004) and 4.3 (p = 0.005) times higher than the daily average during the most recent COP and Earth Day, respectively. At their peak these searches reached the third-highest point ever recorded for climate change or global warming on Google trends.

In addition, DiCaprio claimed that 2015 was the hottest year on record and indigenous populations will be disproportionately affected by climate change. Google queries including the terms “hottest year” or “indigenous” spiked immediately yielding 342% (95%CI: 248–433) and 81% (95%CI: 53–109) more searches than expected the first day following the speech, and remained elevated for 2 and 3 more days, respectively. During the same period, queries for climate change topics not mentioned by DiCaprio, such as “electric cars,” did not deviate from their historical trends.

## Discussion

DiCaprio’s speech was a major moment for climate change advocacy, inspiring record levels of social media engagement and near record levels of online information seeking for climate change, apart from any similar increase in traditional news coverage.

Alongside DiCaprio there are several newly discovered cases showing that organic advocacy—emanating from celebrities and ordinary citizens—have a reach that is greater than virtually any planned media campaign. The most significant smoking-cessation-promoting event in Brazil was when Brazilian President Lula disclosed his laryngeal cancer diagnosis which he attributed to smoking [13]. Angelina Jolie’s editorial in *The New York Times* disclosing her prophylactic double mastectomy corresponded with record levels of news and Google searches for breast cancer, increases that presaged amplified breast cancer screening and prevention [14,15]. Catherine Zeta-Jones disclosed she was treated for bipolar disorder and public engagement with and help-seeking for the condition spiked near record levels [16]. When Charlie Sheen first disclosed his HIV-positive status he personally did not promote HIV prevention, but the public engaged with HIV prevention in record numbers [17]. Ordinary citizen Tawny Willoughby’s Facebook pictures of her skin cancer lesions, attributable to indoor tanning, were shared more than 50,000 times and widely covered by news media [18]. These instances beg the question of how experts might embrace organic advocacy to further their social causes.

Traditional top-down communication strategies—i.e., experts and organizations communicating with the public through planned events and campaigns—have predominated as the method for disseminating scientific evidence and promoting behavior change [19]. These top-down frameworks typically expect that messages are more impactful when shared in the context of a planned campaign [20] and, in some cases, it is almost accepted that scientific evidence is only available and reliably disseminated “from the top.” For instance, many notable reviews, including for climate change communication strategies, do not consider the possibility of information retrieval and sharing outside of the context of a planned campaign [21–23]. However, the example of DiCaprio and others demonstrates that dissemination can occur completely outside the context of a campaign and can even generate more public engagement than planned events. Moreover, in some cases, the scientific community is beginning to find that retaliatory messages from bottom-up communication channels (e.g., on Twitter) can eclipse, dilute, or reduce the efficacy of planned messages [24]. Top-down frameworks are useful but communication strategies should be adapted to include a greater emphasis on bottom-up approaches where experts dedicate more resources to listening, dialoguing, and empowering the public who themselves can become advocates for change.

The use of big media data to inform real time decision-making is an increasingly common phenomenon throughout the sciences [25–28]. To harness the power of big data in the context of scientific communication, developing the infrastructure to detect organic advocacy events virtually in real time should be a priority. As an analogy, the financial industry relies on sophisticated systems for real time decision-making, like the Bloomberg Terminal. These systems map population trends similar to those we outlined here but are available to thousands of users, alerting users automatically to critical deviations in trends, and allowing users to share intelligence with colleagues. It is time the scientific community invest in similar resources. All of the results described herein can be available in real time, including additional forms of passive surveillance and crowdsourced data to understand discrete media events and climate change generally [29–33]. In the case of DiCaprio there was minimal engagement from the scientific community based on our review or related media, likely because experts were not aware of the magnitude of his speech or did not know how to build upon his call for action on climate change.

With awareness of events that can facilitate organic advocacy, the scientific community can engage the public by promoting existing resources and designing new messages targeting public discourse when public engagement and information seeking are high [34]. Given that searches were closely linked to the content of DiCaprio’s speech, the importance of this task is underscored as experts could have filled content gaps and highlighted how the public can mitigate climate change. Reactions could be shared across a diverse array of bottom-up communication channels, such as Facebook, Instagram, Reddit, and Twitter at a very low cost and mirroring how the public is engaged.

By amending our communication frameworks, building infrastructure for monitoring, and reacting to organic advocacy in near real time, we open novel possibilities for communication advocacy and research. For instance, does traditional top-down media advocacy interact with or generate public engagement to discrete organic events? How does organic advocacy about misinformation resonate, such as climate change denial [35] or anti-vaccination [36]? Can events like DiCaprio’s speech drive more than engagement by changing opinions and inspiring action in ways that are complementary to top-down communication approaches [37,38]?

Climate change is a serious crisis with critical implications for public health [39,40]. Climate change advocacy relies on imploring citizens to acknowledge the reality of a warming planet, accept humanity’s role in the fate of the planet, and help to forge a consensus on concrete solutions to the problem [41,42]. DiCaprio issued a wake up call to the public that ignoring climate change poses great peril. At the same time, we believe that ignoring organic advocacy and bottom-up communication approaches imperils the effectiveness of media advocacy and, ultimately, environmental and public health. The scientific community must adapt to the 21st century dynamic communication landscape and ready itself for the next opportunity to harness the agents of change.
